# ‘Essential but not always available when needed’ – an interview study of physicians’ experiences and views regarding use of obstetric ultrasound in Tanzania

**DOI:** 10.3402/gha.v9.31062

**Published:** 2016-07-22

**Authors:** Annika Åhman, Hussein Lesio Kidanto, Matilda Ngarina, Kristina Edvardsson, Rhonda Small, Ingrid Mogren

**Affiliations:** 1 Department of Clinical Sciences, Obstetrics and Gynaecology, Umeå University, Umeå, Sweden; 2 Department of Obstetrics and Gynaecology, Muhimbili National Hospital, Dar es Salaam, Tanzania; 3 Judith Lumley Centre, La Trobe University, Melbourne, VIC, Australia

**Keywords:** obstetric ultrasound, Tanzania, obstetricians, physicians, pregnancy, pregnant women, prenatal diagnosis, qualitative study, fetus, maternal rights

## Abstract

**Background:**

The value of obstetric ultrasound in high-income countries has been extensively explored but evidence is still lacking regarding the role of obstetric ultrasound in low-income countries.

**Objective:**

We aimed to explore experiences and views among physicians working in obstetric care in Tanzania, on the role of obstetric ultrasound in relation to clinical management.

**Design:**

A qualitative study design was applied. Data were collected in 2015, through 16 individual interviews with physicians practicing in obstetric care at hospitals in an urban setting in Tanzania. Data were analyzed using qualitative content analysis.

**Results:**

Use of obstetric ultrasound in the management of complicated pregnancy was much appreciated by participating physicians, although they expressed considerable concern about the lack of ultrasound equipment and staff able to conduct the examinations. These limitations were recognized as restricting physicians’ ability to manage complications adequately during pregnancy and birth. Better availability of ultrasound was requested to improve obstetric management. Concerns were also raised regarding pregnant women's lack of knowledge and understanding of medical issues which could make counseling in relation to obstetric ultrasound difficult. Although the physicians perceived a positive attitude toward ultrasound among most pregnant women, occasionally they came across women who feared that ultrasound might harm the fetus.

**Conclusions:**

There seems to be a need to provide more physicians in antenatal care in Tanzania with ultrasound training to enable them to conduct obstetric ultrasound examinations and interpret the results themselves. Physicians also need to acquire adequate counseling skills as counseling can be especially challenging in this setting where many expectant parents have low levels of education. Providers of obstetric care and policy makers in Tanzania will need to take measures to ensure appropriate use of the scarce resources in the Tanzanian health care system and prevent the potential risk of overuse of ultrasound in pregnancy.

## Introduction

Antenatal care (ANC) is recognized as playing an important role for pregnant women and newborns in low-income countries in improving pregnancy outcomes (1). Many maternal deaths across the world are due to indirect causes such as anemia, malaria, and other pre-existing medical conditions (2). ANC interventions in low-income countries therefore need to be focused on improving maternal health by, for example, managing anemia during pregnancy, preventing and treating malaria, and offering HIV care and prevention (1). Most pregnant women in the sub-Saharan region meet a skilled health provider at least once during pregnancy, and the number of women who attend the four ANC visits or more recommended by the World Health Organization (WHO) is increasing (3), a trend which is paralleled by a decline in maternity mortality (4). Yet, in 2012, only half of all women in the sub-Saharan region attended the four visits for antenatal check-ups. The health care system in this region still faces huge challenges in the provision of maternity care, related to lack of resources such as drugs, equipment, and skilled practitioners (3, 5, 6).

It has been suggested that the offer of a basic routine ultrasound at the lowest level of health care can increase attendance at ANC visits and also motivate pregnant women to deliver at health care facilities (7). In addition, it is argued that the provision of pregnancy ultrasound that includes detailed information of the procedure may mean that pregnant women and medical staff value ANC more highly (8).

Obstetric ultrasound is used extensively across the world and has become a routine part of ANC services in high-income countries. Physicians perceive ultrasound examination as a central tool in obstetric care also in low-income settings (9). Although ultrasound examination can provide benefit to patients when it is included in the ANC program (10), the routine practice of obstetric ultrasound has not yet reached all low-income countries. The value of routine obstetric ultrasound in low-income countries has been debated and there are conflicting views regarding its usefulness in these countries (11). It has been argued that its potential benefits in such areas do not outweigh the costs of routine ultrasound screening in pregnancy (12, 13). Others claim that routine obstetric ultrasound has important benefits in low-income countries, by reducing referrals to tertiary centers for pregnancy surveillance (14) and post-term induction procedures (15). However, it has been recognized that there is a risk of overuse of ultrasound among pregnant women in this setting (9). Ethical concerns have also been raised due to the fact that ultrasound during pregnancy can be used for sex selection of fetuses (16), and that ultrasound has the potential to identify fetal deviations and thereby put increased focus on the health of the fetus. This might affect the pregnant woman's role in decision-making about pregnancy management, such as medical treatment and time and mode of delivery (17).

### Maternal health care in Tanzania

In 2013, Tanzania, with a population of about 45 million, had a Human Development Index ranking of 154 out of 187 countries (18), and is thereby one of the least developed countries in the world. Approximately 70% of the population live in rural areas although rapid urbanization has been noted recently. Although maternal mortality in Tanzania has declined over the recent decades, the maternal mortality ratio of 410 deaths per 100,000 births in 2013 is still high (19). There are large disparities in access to ANC not only between rural and urban areas but also between regions and districts (20). Ultrasound examination during pregnancy has not yet become a routine part of Tanzanian public ANC. Besides larger referral hospitals, most public health care facilities do not have access to ultrasound (pers. comm. Matilda Ngarina 2015). However, pregnant women living in urban areas may attend ultrasound examinations at larger hospitals and private clinics provided they can afford to pay for their examination (pers. comm. Matilda Ngarina 2015).

Pregnant women in Tanzania are positive toward ANC and the majority (>80%) attend ANC at least once, although the quality of care varies (20, 21). ANC in Tanzania must deal with several health challenges such as malaria, HIV/AIDS, and tuberculosis (TB), most often due to HIV co-infection in women (20), which can also negatively affect fetal growth (22). The prevalence of HIV among Tanzanian women aged 15–49 years living in urban areas of the country was 8.9% in 2012 (23). Moreover, maternal health care is also challenged by the high level of illiteracy among women in Tanzania, where in 2010 the rate of illiteracy was 34% in rural areas and 12% in urban areas (23).

### Aim

The aim of this study was to explore the experiences and views of physicians working in obstetric care in Tanzania regarding the role of obstetric ultrasound in the clinical management of pregnancy, and in situations where maternal and fetal health interests conflict.

## Methods

### Study design

This interview study was undertaken by a multidisciplinary research team, representing obstetrics and gynecology, midwifery, nursing, public health research, maternal health research, and epidemiology, as part of the multinational CROss Country Ultrasound Study (CROCUS) (24, 25). A qualitative study design was applied, and data were collected through individual interviews with physicians practicing in obstetric care at hospitals in an urban setting.

### Setting

Three public hospitals providing obstetric care in the Dar es Salaam region were purposively selected for recruitment of participants, and differences were sought regarding the level of care at the hospitals. The three hospitals included one tertiary-level referral hospital that serve both public and private patients, one regional referral hospital, and one municipal hospital. The number of births at the hospitals ranged between 11,000 and 20,000 annually. Most physicians were general practitioners. While the national referral hospital had 20 specialists in obstetrics and gynecology, the regional referral hospital had only four specialists and the municipal hospital had none. All three clinics had at least one ultrasound machine available, but the possibility for a pregnant woman to receive an ultrasound examination free of charge was limited due to the large number of clients. In addition, only a few health care professionals, mainly sonographers, were proficient in using the ultrasound machines. These sonographers were on duty only during daytime, which meant that ultrasound examinations could not be performed during the night unless one of a few trained physicians was available. Public hospitals in the region could charge the private patients 25–60,000 Tanzanian shillings (12–28 USD) for an ultrasound examination and private hospitals even more, around 125,000 Tanzanian shillings (pers. comm. Matilda Ngarina 2015).

### Participant recruitment

Contact was made with the three selected public hospitals in the Dar es Salaam region, and the head of each hospital was asked to assist with the recruitment of participants. Inclusion criteria for participation were being a physician working in antenatal, intrapartum, or postnatal care at a Tanzanian hospital. The physicians practicing in obstetric care available on the day of the interviews were invited to participate. All of the physicians approached agreed to take part in the study.

### Participant characteristics

Sixteen physicians aged between 29 and 52 years (mean 38 years) were recruited. Nine were female and seven were male. Three of the participants were specialists in obstetrics and gynecology, 10 were general practitioners with five years of medical school, and one was a medical intern; two of the participants were assistant medical officers with three years of medical education and training. The participants reported working experience in obstetric care ranging from one month to 22 years (mean five years). A few had experience of caring for women at a private clinic. None of them had participated in any formal education in obstetric ultrasound examinations.

### Data collection

A thematic interview guide developed by the research team and used in all countries participating in the multinational CROCUS was pilot tested in a previous study on Australian obstetricians’ experiences of the significance of obstetric ultrasound for clinical management of complicated pregnancy (24). The guide was used to ensure that all topics were raised during the interviews although not in any predefined order. The key domains in the interview guide are presented in Table 1. The interviewer encouraged participants to elaborate freely on their experiences and views related to the use of obstetric ultrasound.

**Table 1:** Key domains in the CROss Country Ultrasound Study interview guide

Key domains
The importance/value of obstetric ultrasound for clinical management of complicated pregnancy.
The importance of obstetric ultrasound in comparison with other surveillance methods during complicated pregnancy.
Clinical situations where the interests of maternal and fetal health conflict.
Whether the woman may be considered to act as an instrument for fetal treatment.
If/when the fetus can be regarded as a person.
Situations where the fetus has been regarded as a patient with his/her own interests.
Physicians professional role in relation to other occupational groups working with obstetric ultrasound examinations or the outcomes of these examinations.
Other issues in relation to ethical aspects of the use of obstetric ultrasound.

The individual interviews were carried out in February 2015, and took place in separate rooms at the hospital. Before the interview started, the participants completed a questionnaire including questions on age, sex, professional qualifications, and professional experience of obstetrics and obstetric ultrasound examinations. The interviews were conducted in English by IM, AÅ, and KE. All interviews were conducted in a week's time when the authors visited the three hospitals. The local coordinator recruited the participants and after each researcher had conducted one or two interviews, they all met to discuss their initial impressions from their separate interviews, and aspects that were considered important to be further explored were raised in the following interviews. The interviews were digitally recorded and lasted between 15 and 38 min (mean time 26 min).

### Data analysis

Interviews were transcribed verbatim and analyzed using qualitative content analysis (26). First, one member of the research team (AÅ) read all interviews to get a sense of the whole. Data addressing the aims of this study were then coded by AÅ and all coded data were then reviewed by IM. AÅ and IM compared the codes to identify similarities and differences, sorted the codes into content areas, and thereafter categorized the materials into preliminary sub-categories and categories (Table 2). These codes, sub-categories, and categories were then re-reviewed by AÅ and IM, and any diversity in interpretation of the findings was discussed between the two authors until consensus was achieved. An overall theme, three related categories and their eight sub-categories emerged in the analysis. The descriptions of the categories and sub-categories were then reviewed by HLK, MN, KE, and RS, and some additional changes were made. The process from quotes to sub-categories is described in Table 3.

**Table 2:** Theme, main categories, and sub-categories

Theme	Main categories	Sub-categories
Essential but not	I. Ultrasound can enhance pregnancy	Essential in the management of complicated pregnancies
always available	management	Questionable if ultrasound is needed in all pregnancies
when needed	II. Lack of resources limits the availability of	Shortage of usable ultrasound machines
	ultrasound	Physicians should be able to perform ultrasound examinations during pregnancy
	III. Managing pregnant women's different	Many want an ultrasound but not all
	opinions and needs	Adapt counseling based on pregnant women's different needs
		Maternal health should be prioritized but opinions on the fetus diverge
		The woman and her family need to be involved in decision-making

**Table 3:** Examples of quotes, related codes, and sub-categories

Quotes	Codes	Sub-category
*We may find the woman who is bleeding, (…) to be sure you send her very fast to the department of ultrasound so they can give you the result whether it's abruption placenta or it's the placenta previa. So you can very fast and very accurate to take the action*.	Can give fast and accurate results	Essential in the management of complicated pregnancy
*Some women will tell you ‘I have done one ultrasound. I cannot do another one, it is going to harm the baby’*.	Fear that ultrasound can harm the fetus	Many want an ultrasound but not all
*Even the big hospital have deficit of it* [ultrasound]*. Maybe at some* [hospitals] *it was available but then it's out of stock*.	Hospitals have shortage of ultrasound	Shortage of usable ultrasound machines
*We* [the doctors] *would prefer the ultrasound service to be available 24 h in our ward, and prefer that also we working in the department to be trained. It would be much better to be trained to perform ultrasound*.	Physicians want training to be able to perform ultrasound	Physicians should be able to perform ultrasound examinations during pregnancy
*At first* [when malformation is detected] *they are very shocked, they don't know what to do but once you give them the education, (…) they tend to understand and yes they persevere*.	Physicians need to educate women	Adapt counseling based on pregnant women's different needs
*It's better to terminate the pregnancy and save the life of the mother because of that conflict (…) this is a challenge and we have these cases in our hospital and we try to combat this situation of the eclampsia, preeclampsia*.	Most important is to save the life of the mother	Maternal health should be prioritized but opinions on the fetus’ diverge
*We normally try to ask the patient* [about pregnancy management] *but in our tradition the husband will come, the mother in laws and the father maybe, and then you discuss about the matter, then you decide*.	The husband/family members engage in decision-making	The woman and her family need to be involved in decision-making

### Ethical considerations

Ethical approval was obtained from Muhimbili National Hospital Institutional Review Board (reference number MNH/IRB/I/2015/10) and National Institute of Medical Research Review board (reference number NIMR/HQ/R.8a/vol. IX/1985). All participants received written and verbal information about the aims and the procedures of the study, and gave written and verbal consent prior to the interview. The participants were informed that they could withdraw from the study at any time if they wished to do so.

## Results

### Main theme – essential but not always available when needed

The main theme ‘Essential but not always available when needed’ emerged during the analysis. This theme includes three main categories: I. Ultrasound can enhance pregnancy management; II. Lack of resources limits the availability of ultrasound; and III. Managing pregnant women's different opinions and needs, each consisting of two to four sub-categories.

#### Main category I. Ultrasound can enhance pregnancy management

Essential in the management of complicated pregnancy

Obstetric ultrasound was described as the main tool in surveillance of women with complicated pregnancies, and in decision-making regarding the management of such pregnancies. It was greatly valued by the physicians for providing a more accurate diagnosis than clinical examination regarding gestational age, fetal viability, amount of amniotic fluid, placental localization, and fetal presentation. The potential of the ultrasound examination to provide immediate information was perceived as especially valuable as the physicians had a large number of patients needing their attention.

In fact it has got a very big value, first of all when you see the number of the clients who are attending in this area, (…) the number is big, the number of physicians is very little so we need the tool which can simplify the diagnosis. (No. 16)

It was pointed out that results from the ultrasound examination could play a decisive role for management at the time of delivery, especially in cases such as suspected placenta previa or abnormal fetal position. Access to ultrasound was said to be important as ultrasound could confirm the diagnosis and thereby ensure that the physicians take appropriate action directly.

When you are dealing with a scenario where you need to make life changing decisions for the patients, I think it's important to have the ultrasound around, especially when you are dealing with the labour ward. (No. 6)

It was considered troublesome though that ultrasound examination reports from sonographers did not always provide the physicians with accurate information. Some said this could be due to the fact that the ultrasound image was misinterpreted, potentially leading to unnecessary interventions. It was also recognized that both sonographers and physicians sometimes failed to detect fetal deviations on ultrasound examination because of technical problems with the ultrasound machine or insufficient skills of the operator.

Questionable if ultrasound is needed in all pregnancies

Although all participants agreed that ultrasound was highly valuable in the management of complicated pregnancy, there were diverse views regarding whether routine screening ultrasound should be offered to all pregnant women. One of the physicians suggested that every woman should have two screening ultrasound examinations, one in the second trimester to determine gestational age and detect fetal anomalies, and another in the third trimester to prepare for any deviations at birth. Two participants expressed the view that routine ultrasound screening was not necessary at all.

Sometimes you may not need ultrasound because you could see your patient and everything is clear, so you do not need it, but for some patients we need it. So it depends on our client, maybe the condition … they present with. (No. 10)

It was reported that public hospitals provided obstetric ultrasound examinations free of charge, but for medical reasons only, although at times there were no operators present who could perform the examination. Ultrasound examinations were also available, however, at private ANC clinics. Some of the private clinics were situated within the same building as the public hospital facilities and were run by physicians who also worked at the public clinic. Attending an obstetric ultrasound examination at a private clinic meant that the pregnant woman had to pay for the examination. It was also revealed that even when medically indicated, the public hospital could not always provide the women with an ultrasound examination. In such situations, pregnant women could be referred to a private clinic if they had the means to pay for the ultrasound examination.

#### Main category II. Lack of resources limits the availability of ultrasound

Shortage of usable ultrasound machines

Participants reported situations with adverse maternal and fetal conditions such as vaginal bleeding, obstructed labor, and uncertainty regarding viability, where an ultrasound examination was requested but could not be provided because there was no ultrasound equipment that functioned. These public hospitals had only one or two ultrasound machines, and at times these machines were out of order. The high number of patients, recurring power outages, and the lack of air conditioning, which made the room too hot for the ultrasound machine to be used, were factors that further restricted the possibility of obstetric ultrasound examinations.

In most of the hospitals, it's not in every hospital it's accessible, the ultrasound, there are some … even the big hospitals have the deficit of it. Maybe in some it was available but then it's out of stock. (No. 1)

The physicians were very concerned about the fact that they did not always have access to ultrasound in emergency situations, for example, when they needed to locate the position of the placenta or view the fetal presentation. The absence of resources to perform ultrasound was seen as possibly resulting in unnecessary caesarean sections or excessive transfer of patients to more specialized obstetric clinics.

Physicians should be able to perform ultrasound examinations during pregnancy

There were also recurring descriptions in the interviews regarding the lack of staff who were trained to operate the ultrasound machines. At these hospitals, there were only a few specialists in obstetrics who did ultrasound examinations themselves. The most common scenario was physicians sending pregnant women for ultrasound examination to a sonographer who was either a specially trained midwife or radiologist. The people who were trained to do ultrasound examinations commonly worked only during the daytime, which meant that ultrasound examinations could generally not be performed at night.

I was on call at night and there was this woman who was bleeding. I wish I had an ultrasound there and determine what the problem is and see if there is a problem which I can fix, if I should go to the theatre, or I should refer the woman? But I was left there. I didn't know what to do, because the ultrasound person works only during the day so when we are at night we are alone. (No.11)

Now we have obstetric ultrasound but only two people can do it, so if they are not here we can't. (No. 8)

We were donated an ultrasound [*machine*] for us but still we were not trained so we didn't know how to. There is only one person who has been trained (…) she is also in labour ward so, also she doesn't work during the night, so during the night it's the same problem. We have our own ultrasound but still we cannot use it at night. (No. 11)

Although the physicians said that they usually relied on sonographers’ ultrasound assessments, they had experiences of reports from the sonographer not always adequately corresponding with clinical findings.

Sometimes they [*the sonographer*] estimated fetus weight, maybe it's 4.4 kg and you are like no this woman cannot have a vaginal delivery this big baby, and then you go for C Section, you find 3.8 kg or 3.9 kg and you are like, I should have tried vaginal delivery instead. So the information given to you may not be right sometimes. (No. 5)

It was argued that all physicians in obstetric care should be able to perform obstetric ultrasound so that they did not have to rely on others in decision-making regarding pregnancy management. It was recognized, however, that this would require professional teaching and training that currently was absent. It was also pointed out that often the physicians were very occupied with other tasks, which meant that for practical purposes the sonographers performed the ultrasound examinations.

I wish in the future we will get training for us physicians, even nurses, for the basics, at least the basics to know a few things about how to do, how to examine the woman by using ultrasound, or how to determine different things which are important. It would be easier even if you are at night and you have one ultrasound machine here we can use. (No. 11)

One of the physicians reported that the hospital management had planned for physicians to become trained in obstetric ultrasound, but it was not clear when this would happen.

#### Main category III. Managing pregnant women's different opinions and needs

Many want an ultrasound but not all

The physicians reported that most pregnant women presented a positive view toward obstetric ultrasound, and that pregnant women in general perceived ultrasound examinations as more reliable than clinical examinations. They also perceived that the number of pregnant women who requested an ultrasound examination during ANC visits had increased in recent times.

They do know that with ultrasound you can see each and every thing anytime, so the result of ultrasound for them are the definitive and they think they are the best result. (No. 9)

It was also felt that nowadays especially younger and more educated women also wanted to know the sex of the fetus. Some believed that pregnant women wished to know the sex just out of curiosity, or to be able to choose the right colors when buying things for their baby.

It never used to be the case in the past but recently I have noticed that some women would like to know whether they are carrying a baby boy or am I carrying a baby girl? (No. 5)

One of the participants, however, stated that some pregnant women had a preference for boys and that they might want to undergo an ultrasound examination to be able to terminate the pregnancy if it was a female fetus.

There is this stigma between girls and boys, in some communities they want to know if it's a boy or a girl so that they may be able to either prevent the pregnancy from going on. (No. 6)

Although the physicians found that pregnant women often were very excited about having an ultrasound examination and the possibility to view the fetus, some thought that there were pregnant women who did not show any interest at all in the ultrasound examination.

It is not until recent that people came to know about the ultrasound in this country, in our setting, so now that we know about it people are more enthusiastic about the ultrasound and (…) there are people who will agree that the ultrasound has a positive impact. Others will say no because others still believe that the pregnancy should go on in the natural outcome, we should not intervene by seeing what's going on in there, but those people are very few compared to those who do want to know how the baby is doing. (No. 6)

Moreover, it was also mentioned that a few pregnant women were fearful that the ultrasound examination would harm their fetus. Further, it was pointed out that when pregnant women had to pay for the ultrasound they might refrain from having the examination because they felt it was too expensive and not worth it, or because they just could not afford it.

Adapt counseling based on pregnant women's different needs

The physicians reported that pregnant women's level of knowledge and understanding regarding the pregnancy ultrasound varied greatly, and that this sometimes made counseling about the results of the ultrasound examination complicated. Thus, physicians needed to adapt their information and counseling to the woman's individual level of understanding.

Because you are dealing with women with different education and different understanding so you just decide according to the level of understanding of the patient. (No. 1)

The physicians commented that pregnant women rarely requested any detailed information about the ultrasound findings but merely wanted them to confirm that everything was well with their fetus. It was also mentioned that it had become more common lately that pregnant women asked questions about the fetus’ health.

They want to know is the baby okay?! Is it kicking?! It's those little, little, little issues, that's all. (No 5)

Maternal health should be prioritized but opinions about the fetus diverge

Although ultrasound was said to be very helpful in many cases, it was recognized that the potential of ultrasound to assess fetal viability could bring into light conflicting interests between the health of the mother and the health of the fetus regarding pregnancy management.

If it is towards near [*the gestational age of*] viability of the fetus we try as much as it's possible to compromise it too. Like people with say breast cancer, which comes onto 26 weeks 28 then you try to push a little bit so that you deliver a viable baby, but at times we decide to forsake the baby. (No. 2)

It's difficult …, it's better to terminate the pregnancy and saving the life of the mother because of that conflict, as we said that this is a challenge, and we have these cases in our hospital and we try to combat them, this situation of the eclampsia, preeclampsia. (No. 7)

On rare occasions, physicians had faced situations where they could not intervene to save the fetus without risking the health of the pregnant woman. It was claimed that in such cases, the well-being of the pregnant women was their first priority.

When we have the eclampsia, the interest is the mother and we don't consider the fetus anymore even if …. the survival are there but first it should be the mother because this is really a person that we are sure we need. (No. 1)

There were divergent opinions among the physicians as to whether the fetus could be regarded as a person or not, and to what extent the health of the fetus should impact on pregnancy management. Some physicians perceived the fetus as a person from the time of viability, while others thought that the fetus should be perceived as a person from conception. It was experienced also that pregnant women recognized the fetus as a human being when they saw the ultrasound image.

I think it [*the ultrasound image*] will affect them [*the pregnant women*] because they know it's really a person and so it is moving, it is breathing, and there is heartbeat and there is swallowing so I think that it will change them. (No. 11)

In addition, physicians had experiences of decision-making being complicated, and that sometimes they postponed delivery for the sake of the fetus although the pregnant woman's health might be at risk. One of the physicians argued that it could sometimes be justified to let the women take such risks as one could not be sure that future pregnancies would turn out well.

Other physicians claimed that they had never experienced a mother being sacrificed for the sake of the fetus. Further, it was pointed out that the possibility to treat fetal conditions was very limited in this setting.

The woman and her family need to be involved in decision-making

The physicians said it was important that pregnant women expressed their own opinions during counseling about ultrasound findings and pregnancy management. It was recognized however, that physicians also might have to discuss management with family members when there were medical issues regarding the pregnancy.

Most of them, I have seen both of them [the pregnant woman and her husband] talk which is good, a third of them it's the man that says everything and the lady just says it's okay, but in most of my conversation I have seen the men, both of them talk and they agree on something, and yes we plan for the next management. (No. 4)

Although it was common that the family agreed to the physician's suggestion on management, there were also situations described where pregnant women suffered from pre-eclampsia in the second trimester of pregnancy and their relatives had said no to termination of pregnancy although the woman's health was at stake.

She may come earlier with more problems so we try if we can save the fetus. If it's far-fetched then we terminate the pregnancy but even termination is something else because if the important others may say no. (No. 2)

The physicians felt that pregnant women could also become very worried when they were informed about deviating ultrasound findings, even when the findings had little significance for pregnancy management, such as, for example, detection of breech presentation in the second trimester. It was mentioned, however, that proper counseling concerning ultrasound findings and pregnancy management might be overlooked because of the large number of patients that the physicians had to attend to.

## Discussion

In this study, we explored physicians’ experiences and views on the use of obstetric ultrasound in an urban setting in Tanzania. The results from these interviews show that the use of ultrasound in management of complicated pregnancy was greatly valued. There were considerable concerns though, regarding the shortage of ultrasound equipment and lack of skilled health professionals to manage the ultrasound machines at the clinics. The lack of resources was said to restrict the possibility of conducting ultrasound examinations, sometimes also in situations where an ultrasound could be essential for adequate decision-making regarding pregnancy management. It was stressed that more physicians in maternity care should be trained so that they could perform ultrasound during pregnancy themselves and thereby make ultrasound more available in obstetric care.

Obstetric ultrasound is known to be valued by health professionals in obstetric care (24), something which our results also confirm in this low-income setting. The benefit of second trimester ultrasound screening in low- and middle-income countries has, however, been debated, as routine scans do not seem to be associated with any reduction in adverse outcomes for babies, or decrease in the use of health services by mothers and babies (27, 28). The value of ultrasound examinations in management of complicated pregnancy has not been questioned (29). It has also been shown that ultrasound examinations in late pregnancy have the potential to improve pregnancy management in developing countries especially when the decision to do an ultrasound examination is based on preceding risk stratification (29). Our findings revealed a substantial deficiency regarding access to ultrasound in the study setting, a deficiency that was related both to the health professionals’ own lack of training as well as to a shortage of working ultrasound equipment in these public hospitals. Although private clinics in the same area could offer obstetric ultrasound examinations, this possibility was restricted to patients able to pay for their examination. If a situation persists wherein public hospitals cannot provide pregnant women with an ultrasound examination free of charge, the financial barriers can mean that pregnant women refrain from having an obstetric ultrasound despite strong medical indications. Apart from economic barriers, it is known that pregnant women in Tanzania refrain from visits for antenatal check-ups to hospitals or health care centers for practical reasons such as travel distance and waiting time (30).

In the future, ultrasound equipment may become more available as lightweight portable ultrasound devices that are less expensive have been introduced. It is suggested that this development may mean that ultrasound will be more available also in low-resource settings such as Tanzania (31). It is emphasized though that lack of adequate training can be a barrier for health care professionals in developing countries to properly use ultrasound in obstetric care (6). In addition, poor interpretation of the ultrasound image can result in inadequate counseling and unnecessary intervention such as, for example, caesarean section. Further, it is of great importance to avoid unnecessary caesarean sections in the Tanzanian setting where complications related to this intervention are known to cause life-threatening conditions and even maternal deaths (32). It has been noted that many practitioners conducting ultrasound examinations in low- and middle-income countries require more training in the performance of the examination to meet WHO criteria regarding the length of the training program (33). However, it is stressed that investments in obstetric ultrasound in low-income counties should be balanced with other needs in maternity care, as many maternal deaths are attributed to causes such as hemorrhage, hypertensive disorders, and sepsis (2). Moreover, the number of skilled health care professionals required in the Tanzanian health care system by far exceed the number of health care professionals available (34). It is suggested that this situation is mainly due to the increasing number of people living with HIV/AIDS (34). Therefore, careful weighing up of the distribution of resources in the health care system in Tanzania is needed.

Our results point to a growing interest in ultrasound examinations among pregnant women in Tanzania, especially among more educated women. Pregnant women who receive an ultrasound examination have shown a high level of satisfaction (35). However, the current study has revealed that some pregnant women are also fearful that the ultrasound examination could harm the fetus. It has been found earlier that pregnant women in Tanzania who have this fear might still attend the examination when it is offered believing the scan to be obligatory (36). Appropriate information and communication with pregnant women regarding benefits and limitations of obstetric ultrasound has been suggested to relieve fear and also prevent irrational expectations and demands (11). Still, counseling concerning ultrasound findings can be hampered by lack of ultrasound education and training among health care professionals. Further, antenatal health care professionals need to be aware of the risk that pregnant women might overestimate the significance of the ultrasound (9), and that professionals also might overuse obstetric ultrasound at the expense of a thorough recording of medical history and physical examinations (11).

The technical development of the ultrasound and the increasing possibility to treat fetal conditions have been recognized as sometimes entailing complex ethical dilemmas when an intervention for the sake of the health of the fetus conflicts with the health interest of the pregnant woman (37). Concerns have been raised about pregnant women's position in decision-making regarding management of pregnancy (38), which might be especially the case for pregnant women in low-income settings where many women have little or no education, and where the illiteracy rate is high (23). Pregnant women's rights to autonomous decision-making have been reported to be inconsistently supported by ANC professionals even in high-income settings like Australia (39). In addition, the use of ultrasound to determine fetal sex has raised concerns regarding the risk of selective abortions as a result of expectant parents’ sex preference of their child (40).

### Strengths and limitations of the study

In this multidisciplinary and multinational research group, there were two researchers from Tanzania who were familiar with the local context, as well as one researcher from Sweden who had previously been involved in the Tanzanian health care system and culture, strengthening the conduct and interpretation of this study. Our results are also strengthened by the purposive sampling employed, that is, we recruited participants who were expected to provide information that could answer our research questions in the best way.

The participants were of different ages, genders, and had different levels of post graduate education and years of professional experience in obstetric care, all of which likely will enhance transferability of the results. Only three of the participants were specialists in obstetrics and gynecology, and they all worked at the national referral hospital. However, in the study setting, most doctors were general practitioners. While the national referral hospital had 20 specialists in obstetrics and gynecology, the regional referral hospital had only 4 specialists and the municipal hospital had none. The transferability is also limited to the urban low-income setting and the local health care system.

All key domains were raised in each interview and the data obtained were considered very rich. Some interviews were short as the participants did not express their experience at length and were limited by time. During the week the data were collected, the three researchers conducting the interviews met several times to share their impressions from their interviews. In addition, results from the analysis were discussed with the other researchers in the team, something which further enhanced reflexivity.

The interview guide used in this study was developed for use in a variety of settings and cultures in both high-income and low-income countries. This means that some of the topics included in the interview guide were of limited relevance to the Tanzanian setting, such as the question whether the woman may be considered to act as an instrument for fetal treatment, given so little fetal treatment is currently possible in Tanzania.

## Conclusions

Obstetric ultrasound plays a significant role in the medical management of complicated pregnancy and childbirth. This interview study showed a great appreciation of pregnancy ultrasound among the participating physicians but also concerns about deficiencies in terms of ultrasound equipment and the number of physicians proficient in performing the examinations and interpreting ultrasound images on their own. There seems to be a need to provide more physicians in ANC in Tanzania with training in using ultrasound to enable them to conduct obstetric ultrasound examinations and interpret the results themselves. Further, this will entail a need also for physicians to acquire adequate counseling skills as counseling pregnant women and their relatives involved in decision-making can be especially challenging in this setting where many expectant parents have low levels of education. With the increasing use of pregnancy ultrasound, providers of obstetric care and policy makers in Tanzania will need to take measures to ensure the appropriate use of the scarce resources in the Tanzanian health care system and prevent the potential risk of overuse of ultrasound in pregnancy, as well as the risk that ultrasound will be used for fetal sex selection, as already reported in some low-income countries (41).

## Paper context

Obstetric ultrasound plays a significant role in the management of complicated pregnancy and childbirth also in low income countries. This study shows a great appreciation of obstetric ultrasound among the Tanzanian physicians but also concerns about deficiencies in terms of ultrasound equipment and number of physicians proficient in performing the examinations. With increasing use of pregnancy ultrasound measures need to be taken to ensure appropriate use of the scarce resources in the Tanzanian health care.
